# Early mortality in lung cancer: French prospective multicentre observational study

**DOI:** 10.1186/s12890-016-0205-5

**Published:** 2016-04-02

**Authors:** Michel Grivaux, Didier Debieuvre, Dominique Herman, Christine Lemonnier, Jean-Michel Marcos, Jacky Crequit, Sylvie Vuillermoz-Blas, Patricia Barre, Marie Saillour, Francis Martin

**Affiliations:** Service de pneumologie, Centre hospitalier, BP 218, 6-8 rue Saint Fiacre, Meaux, 77104 Cedex France; Service de pneumologie, Hôpital Emile Muller, Mulhouse, France; Service de pneumologie, Hôpital Pierre Beregovoy, Nevers, France; Service de pneumologie, Centre hospitalier, Auxerre, France; Service de pneumologie, Centre hospitalier, Libourne, France; Service de pneumologie, Hôpital Laënnec , Creil, France; Service de pneumologie, Hôpital St-Joseph-St-Luc , Lyon, France; Service de pneumologie, Centre hospitalier, Cahors, France; Service de pneumologie, Centre hospitalier, Nanterre, France; Pneumologie et Pathologies du sommeil, Centre hospitalier Intercommunal Compiègne-Noyon, Compiègne, France

**Keywords:** Epidemiology, France, Hospital, KBP-2010-CPHG, Lung neoplasms, Mortality

## Abstract

**Background:**

Despite the progress seen in the last decade in diagnosis and treatment, lung cancer has still a bad prognosis and a substantial number of patients died within the weeks following diagnosis. The objective of this study was to quantify early mortality in lung cancer, to identify patients who are at high risk of early decease, and to describe their management in a real world.

**Methods:**

Prospective observational study including consecutively all adult patients managed for primary lung cancer histologically or cytologically diagnosed in 2010 in the respiratory medicine department of one of the participating French general hospitals. Patients and cancer characteristics and first therapeutic strategy were collected at diagnosis. Dates of death were obtained from investigators or town council of the patient’s birth place. All fatal cases were considered regardless of the cause of the death. Multivariate logistic regression model was used to determine the factors significantly and independently associated with death at 1 and 3 months.

**Results:**

Seven thousand fifty-one patients from 104 centres were included in the study. Vital status was obtained for 6,981 patients. Respectively, 678 (9.7 %) and 1,621 (23.2 %) of the 6,981 patients with available data died within 1 and 3 months following diagnosis. As compared with the other patients, they were significantly older and frailer (based on performance status [PS] and recent weight loss) and more frequently reported stage IV tumour. Overall, 64.5 % (1 month) and 42.8 % (3 months) of patients had no cancer therapy and less than 1 % were included in a therapeutic trial.

**Conclusion:**

About one in four patients died within 3 months following lung cancer diagnosis. Early mortality mainly involves frail patients with advanced cancer and is associated with lack of cancer therapy. This supports the need for early diagnosis and clinical trials in this population. Reducing early mortality to give supplementary time to patients to organise the future is a major challenge for 21^st^ century physicians.

## Background

Despite the progress seen in the last decade in diagnosis and treatment, lung cancer has still a bad prognosis [1]. In France, in 2010, 1-year mortality rate in patients with lung cancer was estimated at 56 % [2].

Literature on lung cancer mortality is abundant. Between 01-January-2000 and 30-November-2015, more than 8,000 articles with an abstract could be identified when consulting PubMed with the following sentence: ‘lung neoplasms [Mesh]’ and ‘mortality [Mesh]’. However, available information about early mortality is limited [3]. Most of the literature relates to treatment and usually excludes elderly, frail, and socially marginalised patients [3], or tries to identify predictors of early mortality after specific therapeutic interventions (e.g., surgical resection) or in specific populations (e.g., patients with comorbidity).

In 2010, the French College of General Hospital Respiratory Physicians (CPHG) conducted a prospective observational study, KBP-2010-CPHG, whose main objective was to assess 5-year mortality rate. This study involved the respiratory medicine department of 119 French general hospitals. Overall, 7,610 adults managed with primary lung cancer histologically or cytologically diagnosed between the 01-January-2010 and 31-December-2010 in the participating respiratory medicine departments were included [4].

As a significant proportion of patients from the KBP-2010-CPHG died just after the diagnosis of lung cancer, a *post hoc* analysis was performed to (a) quantify early mortality (1 and 3 months), (b) to identify the main characteristics of patients who early died, and (c) to describe their management. From health care system and professional perspectives, identifying patients who early died and describing their management emphasize the need for earlier diagnosis and palliative approaches and for more effective treatment. From a patient or family perspectives, they emphasize the need for more effective treatment and improved support to save time and better face this unfavourable outcome [5].

## Methods

KBP-2010-CPHG is a French prospective multicentre observational study. The study protocol was approved by the advisory committee on research information processing in the health field (CCTIRS) on 19 November 2009 and the French Information technology and freedoms commission (CNIL) on 11 January 2010 (909479). The ethics committee of the French Speaking Society of Pneumology confirmed the observational nature of the study on 23 April 2010 (N°2010-008). All included patients were duly informed of the study objectives and requirements, and gave their oral consent before inclusion.

At the end of 2009, the members of the CPHG which gathers the chest physicians of the respiratory departments of the French general hospitals (overseas departments and territories included) were contacted. Those agreeing to participate became study investigators and their respiratory medicine department a study centre. Each investigator has to exhaustively include all patients aged over 18 years managed in his/her respiratory medicine department for a primary lung cancer histological or cytological diagnosed with a sample collected between 1^st^ January and 31^st^ December 2010. The date of sample was considered to be the date of the diagnosis. Then, the investigator filled out an anonymous questionnaire comprising items on the patient, his/her tumour, and its treatment really performed. A steering committee assessed study compliance [4, 6]. Vital status and date of death were obtained by the investigator or town council of the patient’s birth place at least 1-year after the date of diagnosis.

All fatal cases were considered regardless of the cause of the death. Patients who were alive 1 and 3 months after the diagnosis date were censured at this date. First, data were described using mean and standard deviation (mean [SD]) or frequency and percentage (number [%]). At 1 and 3 months, Chi^2^-test or Fisher test (when Chi^2^-test criteria were not respected) and Student *t* test and analysis of variance (ANOVA) (for normal distributions) or non-parametric tests (for non-normal distributions) were used to compare died and alive patients in univariate analyses. A multivariate logistic regression model was used to determine the factors significantly and independently associated with death at 1 and 3 months. Statistical test results were considered significant at *p < 0.05* (2-sided). Missing data were not replaced.

## Results

### Study follow-up and baseline characteristics of the study population

A total of 119 respiratory medicine departments participated in the study and included 7,610 patients. Fifteen (15) centres deemed to be non-exhaustive by the steering committee were excluded from the study analysis. In addition, some patients were excluded from the study as they presented with major protocol failures: i.e., no information on histological or cytological sampling, sampling date outside of the recommended window, primary lung cancer not confirmed at histology, or cancer managed outside of the study centre. Finally, the KBP-2010-CPHG analysis population included 7,051 patients (including 6,083 patients with non-small-cell lung cancer [NSCLC]) from 104 centres.

### Mortality rates within the 1^st^ year following diagnosis

The vital status at 1 year and the date of death (for died patients) were obtained for 6,981 patients. Respectively, 678 (9.7 %) and 1,621 (23.2 %) patients died within 1 and 3 months following diagnosis.

### Characteristics of the patients according to their vital status at 1 and 3 months

Main characteristics of patients according to their vital status 1 and 3 months following diagnosis are presented in Table 1.

**Table 1:** Characteristics of the patients (*N* = 6,981) according to vital status^a^ (*N* = 6,981)

		Vital status at					
		1 month			3 months		
		Alive	Dead		Alive	Dead	
	*N*	*N* = 6,303	*N* = 678	*p-value*	*N* = 5,360	*N* = 1,621	*p-value*
Sex		***n*** **= 6,303**	***n*** **= 678**	***0.018***	***n*** **= 5,360**	***n*** **= 1,621**	*0.097*
Men, n (%)	5,286	4,747 (75.3)	539 (79.5)		4,033 (75.2)	1,253 (77.3)	
Women, n (%)	1,695	1,556 (24.7)	139 (20.5)		1,327 (24.8)	368 (22.7)	
Age		***n*** **= 6,303**	***n*** **= 678**	***<0.001***	***n*** **= 5,360**	***n*** **= 1,621**	***<0.001***
≤40 years, n (%)	73	66 (1.1)	7 (1.0)		61 (1.1)	12 (0.7)	
41-50 years, n (%)	536	502 (8.0)	34 (5.0)		448 (8.4)	88 (5.4)	
51-60 years, n (%)	1,876	1,730 (27.5)	146 (21.5)		1,519 (28.3)	357 (22.0)	
61-70 years, n (%)	2,053	1,865 (29.6)	188 (27.7)		1,612 (30.1)	441 (27.2)	
71-80 years, n (%)	1,713	1,530 (24.3)	183 (27.0)		1,260 (23.5)	453 (28.0)	
>80 years, n (%)	730	610 (9.7)	120 (17.7)		460 (8.6)	270 (16.7)	
Age (years)		***n*** **= 6,303**	***n*** **= 678**	***<0.001***	***n*** **= 5,360**	***n*** **= 1,621**	***<0.001***
Mean (SD)		65.2 (11.2)	68.2 (11.7)		64.7 (11)	68.2 (11.7)	
Median (Q1-Q3)		65 (57-74)	68 (60-78)		64 (57-73)	68 (60-78)	
Body mass index (BMI; kg/m^2^)		***n*** **= 5,974**	***n*** **= 558**	***<0.001***	***n*** **= 5,139**	***n*** **= 1,393**	***<0.001***
Mean (SD)		24.4 (4.8)	23.5 (4.8)		24.6 (4.8)	23.3 (4.7)	
Median (Q1-Q3)		23.9 (21-27.1)	23.2 (20.3-26.3)		24.1 (21.2-27.3)	22.9 (20.1-26.1)	
Weight loss within the last 3 months		***n*** **= 6,154**	***n*** **= 625**	***<0.001***	***n*** **= 5,249**	***n*** **= 1,530**	***<0.001***
No, n (%)	3,143	2,985 (48.5)	158 (25.3)		2,717 (51.8)	426 (27.8)	
Yes, n (%)	3,636	3,169 (51.5)	467 (74.7)		2,532 (48.2)	1,104 (72.2)	
If yes		***n*** **= 3111**	***n*** **= 446**	***<0.001***	***n*** **= 2,493**	***n*** **= 1,064**	***<0.001***
<5 kg, n (%)	1,489	1,340 (43.1)	149 (33.4)		1,141 (45.8)	348 (32.7)	
5-10 kg, n (%)	1,452	1,270 (40.8)	182 (40.8)		1,005 (40.3)	447 (42.0)	
≥10 kg, n (%)	616	501 (16.1)	115 (25.8)		347 (13.9)	269 (25.3)	
Performance status at diagnosis		***n*** **= 6,235**	***n*** **= 672**	***<0.001***	***n*** **= 5,299**	***n*** **= 1,608**	***<0.001***
0- Fully active, n (%)	1,885	1,855 (29.8)	30 (4.5)		1,765 (33.3)	120 (7.5)	
1- Restricted in heavy physical work, n (%)	2,872	2,749 (44.1)	123 (18.3)		2,462 (46.5)	410 (25.5)	
2- Up and about more than half the day, n (%)	1,273	1,103 (17.7)	170 (25.3)		812 (15.3)	461 (28.7)	
3- In bed or sitting in a chair more than half the day, n (%)	685	460 (7.4)	225 (33.5)		231 (4.4)	454 (28.2)	
4- In bed or in a chair all the time, n (%)	192	68 (1.1)	124 (18.5)		29 (0.6)	163 (10.1)	
Smoking status		***n*** **= 6,271**	***n*** **= 668**	*0.831*	***n*** **= 5,335**	***n*** **= 1,604**	*0.803*
Never-smoker, n (%)	752	679 (10.8)	73 (10.9)		583 (10.9)	169 (10.5)	
Former-smoker, n (%)	2,776	2,516 (40.1)	260 (38.9)		2,124 (39.8)	652 (40.7)	
Active-smoker, n (%)	3,411	3,076 (49.1)	335 (50.2)		2,628 (49.3)	783 (48.8)	
Tobacco consumption (pack-years)^b^	**5,893**	***n*** **= 5,352**	***n*** **= 541**	***0.003***	***n*** **= 4,566**	***n*** **= 1,327**	***<0.001***
Mean (SD)		42.8 (21.3)	45.3 (22.4)		42.5 (21)	44.9 (22.8)	
Median (Q1-Q3)		40 (30-50)	40 (30-55)		40 (30-50)	40 (30-54)	
Tobacco duration (years)^b^	**5,220**	***n*** **= 4,763**	***n*** **= 457**	***0.015***	***n*** **= 4,067**	***n*** **= 1,153**	***<0.001***
Mean (SD)		37.4 (11.6)	38.7 (11.5)		37.2 (11.6)	38.5 (11.6)	
Median (Q1-Q3)		40 (30-45)	40 (30-46)		39 (30-45)	40 (30-45)	

^a^1 and 3 months after the diagnosis of primary lung cancer (date of diagnosis = date of histological or cytological sampling);

^b^Smokers (former or active) only

N or n: number of subjects; Q1-Q3: First and third quartiles; SD: standard deviation

Note: Significant *p*-value are in bold

Compared with other patients, patients who early died were older (*p* < 0.001), although 7 (9.6 %) and 12 (16.4 %) of the 73 patients aged 40 years or less died within 1 and 3 months following diagnosis, respectively.

Patients who early died were leaner than the other patients (*p* < 0.001) and had more frequently lost weight within the 3 months preceding the diagnosis (*p* < 0.001). Of the 616 patients having lost 10 kg or more, 115 (18.7 %) and 269 (43.7 %) died within 1 and 3 months.

Compared with other patients, patients who early died more frequently had a poorer performance status (PS ≥ 2) (*p* < 0.001).

No significant differences was observed between early dead patients and other patients in smoking status (*p* = 0.831 and *p* = 0.803 at 1 and 3 months, respectively), but among smokers (former or active), heavier consumer tended to die prematurely (*p* = 0.003 and *p* < 0.001, respectively).

### Characteristics of the tumours according to patient’s vital status at 1 and 3 months

Table 2 presents the main characteristics of the tumour according to patients’ vital status 1 and 3 months following diagnosis.

**Table 2:** Characteristics of the tumour according to patients’ vital status^a^ (*N* = 6,981)

		Vital status at					
		1 month			3 months		
		Alive	Dead		Alive	Dead	
	*N*	*N* = 6,303	*N* = 678	*p-value*	*N* = 5,360	*N* = 1,621	*p-value*
Histology		***n*** **= 6,303**	***n*** **= 678**		***n*** **= 5,360**	***n*** **= 1,621**	
Small-cell carcinoma, n (%)	961	829 (13.2)	132 (19.5)	***<0.001***	725 (13.5)	236 (14.6)	*0.309*
Adenocarcinoma, n (%)	3,221	2,944 (46.7)	277 (40.9)	***0.004***	2,482 (46.3)	739 (45.6)	*0.632*
Squamous-cell carcinoma, n (%)	1,875	1,733 (27.5)	142 (20.9)	***<0.001***	1,488 (27.8)	387 (23.9)	***0.002***
Large-cell carcinoma, n (%)	786	665 (10.6)	121 (17.9)	***<0.001***	544 (10.2)	242 (14.9)	***<0.001***
Adenocarcinoma *in situ*, n (%)	77	72 (1.1)	5 (0.7)	*0.444*	67 (1.3)	10 (0.6)	***0.045***
Carcinoid tumour, n (%)	40	39 (0.6)	1 (0.2)	*0.176* ^b^	38 (0.7)	2 (0.1)	***0.011***
Other, n (%)	111	102 (1.6)	9 (1.3)	*0.679*	86 (1.6)	25 (1.5)	*0.095*
Genomic mutation		***n*** **= 6,237**	***n*** **= 670**		***n*** **= 5,301**	***n*** **= 1,606**	
Explored, n (%)	2,111	1,969 (31.6)	142 (21.2)	***<0.001***	**1,685 (31.8)**	426 (26.5)	***<0.001***
If explored,		***n*** **= 1806**	***n*** **= 121**		***n*** **= 1,555**	***n*** **= 372**	
EGFR mutated, n (%)	202	196 (10.9)	6 (5.0)	*0.058*	179 (11.5)	23 (6.2)	***0.003***
Stage (7 edition)		***n*** **= 6,264**	***n*** **= 672**	***<0.001***	***n*** **= 5,334**	***n*** **= 1,602**	***<0.001***
Stage ≤ IIB, n (%)	1,129	1,100 (17.6)	29 (4.3)		1,056 (19.8)	73 (4.6)	
Stage IIIA, n (%)	934	905 (14.4)	29 (4.3)		845 (15.8)	89 (5.6)	
Stage IIIB, n (%)	705	643 (10.3)	62 (9.2)		571 (10.7)	134 (8.4)	
Stage IV, n (%)	4,168	3,616 (57.7)	552 (82.1)		2,862 (53.7)	1,306 (81.5)	

^a^1 and 3 months after the diagnosis of primary lung cancer (date of diagnosis = date of histological or cytological sampling);

^b^Fisher test (violation of Chi² test conditions)

N or n: number of subjects

Note: Significant *p*-value are in bold

Compared with other patients, patients who early died more frequently had small-cell lung cancer (SCLC) at 1 month (19.5 % versus 13.2 %, *p* < 0.001; mortality rate: 13.7 %) but not at 3 months (14.6 % versus 13.5 %, *p* < 0.001; mortality rate: 24.6 %). Compared with other patients with NSCLC, patients who early died more frequently had large-cell carcinoma at 1 month (17.9 % versus 10.6 %, *p* < 0.001; mortality rate: 15.4 %) and at 3 months (10.2 % versus 14.9 %, *p* < 0.001; mortality rate: 30.8 %). They less frequently had adenocarcinoma, squamous-cell carcinoma, or other lung cancer.

EGFR-mutation tests were performed for 2,111 patients, mainly patients who did not early died (*p* < 0.0001). When explored, EGFR-mutation was less frequently reported in early died patients than in the other patients (*p* = 0.058 at 1 month and *p* = 0.003 at 3 months). The tumour of 6 (3.0 %) and 23 (11.4 %) of the 202 explored patients died within 1 and 3 months following diagnosis carried the EGFR mutation.

Stage IV tumour was more frequent in patients who early died than in the other patients. Respectively, 29 (2.6 %) and 73 (6.5 %) of the 1,129 patients with lung cancer of stage IIB and over died within 1 and 3 months following diagnosis.

### Independent risk factors of early death

Multivariate analysis (Table 3) confirmed that impaired performance status (PS > 0) (PS4: OR = 71.9 [41–130.28], *p* < 0.001, at 1 month and OR = 44.23 [26.29–77.66], *p* < 0.001, at 3 months), advanced cancer (OR = 2.17 [1.24–3.88], *p* = 0.008 and OR = 2.36 [1.49-3.92], *p* < 0.001, at 1 month and OR = 2.44 [1.68–3.56], *p* < 0.001 and OR = 3.66 [2.72–5.02], *p* < 0.001, at 3 months, for stage IIIB and stage IV, respectively), weight loss within the 3 months preceding diagnosis (OR = 1.4 [1.09–1.81, *p* < 0.01 at 1 month and OR = 1.54 [1.3–1.82], *p* < 0.001 at 3 months), and large-cell carcinoma (OR = 1.72 [1.23–2.38], *p* = 0.001, at 1 month and OR = 1.33 [1.04–1.68], *p* = 0.022, at 3 months) were independent risk-factors of 1 and 3-month mortality. Small-cell carcinoma was an independent risk-factor of mortality at 1 month (OR = 1.39 [1.03–1.87], *p* = 0.032) but a protective-factor at 3 months (OR = 0.73 [0.58–0.92], *p* = 0.008). Finally, multivariate analysis showed that female gender (OR = 0.69 [0.52–0.9], *p* = 0.007) was an independent protective-factor of 1-month mortality and old age (>70 years) (OR = 1.51 [95 % CI: 1.11–2.08], *p* = 0.011) an independent risk-factor of 3-month mortality.

**Table 3:** Independent significant risk factors of death^a^ (*N* = 5,548) – Reduced model

		1 month			3 months		
		OR	95 % CI		OR	95 % CI	
	*N*		[Lower bound; Upper bound]	*p-value*		[Lower bound; Upper bound]	*p-value*
Age							
≤50 years					1		
51-70 years					1.18	[0.89; 1.59]	*0.267*
>70 years					1.51	[1.11; 2.08]	***0.011***
Sex							
Men	4,179	1			1		
Women	1,369	0.69	[0.52; 0.9]	***0.007***	0.86	[0.71; 1.04]	*0.129*
Weight loss within the 3 preceding months							
No	2,545	1			1		
Yes	3,003	1.4	[1.09; 1.81]	***0.01***	1.54	[1.3; 1.82]	***<0.001***
Smoking status							
Never-smoker					1		
Former-smoker					1.38	[1.05; 1.83]	***0.024***
Active-smoker					1.36	[1.03; 1.81]	***0.031***
Performance status at diagnosis							
0- Fully active	1,576	1			1		
1- Restricted in heavy physical work	2,362	2	[1.27; 3.28]	***0.004***	1.82	[1.43; 2.34]	***<0.001***
2- Up and about more than half the day	1,002	6.5	[4.14; 10.63]	***<0.001***	5.07	[3.92; 6.6]	***<0.001***
3- In bed or sitting in a chair more than half the day	496	20.2	[12.84; 33.15]	***<0.001***	17.27	[12.93; 23.25]	***<0.001***
4- In bed or in a chair all the time	112	71.9	[41; 130.28]	***<0.001***	44.23	[26.29; 77.66]	***<0.001***
Stage (7 edition)							
Stage ≤ IIB	901	1			1		
Stage IIIA	759	0.75	[0.38; 1.46]	*0.397*	1.17	[0.79; 1.74]	*0.436*
Stage IIIB	570	2.17	[1.24; 3.88]	***0.008***	2.44	[1.68; 3.56]	***<0.001***
Stage IV	3,318	2.36	[1.49; 3.92]	***<0.001***	3.66	[2.72; 5.02]	***<0.001***
Histology							
Adenocarcinoma	2,521	1			1		
Small-cell carcinoma	758	1.39	[1.03; 1.87]	***0.032***	0.73	[0.58; 0.92]	***0.008***
Squamous-cell carcinoma	1,440	0.89	[0.66; 1.2]	*0.454*	0.91	[0.75; 1.11]	*0.348*
Large-cell carcinoma	586	1.72	[1.23; 2.38]	***0.001***	1.33	[1.04; 1.68]	***0.022***
Adenocarcinoma *in situ*	48	2.05	[0.45; 6.43]	*0.275*	1.59	[0.62; 3.64]	*0.298*
Carcinoid tumour	78	1.01	[0.36; 2.45]	*0.983*	1.01	[0.5; 1.95]	*0.983*
Other	117	1.44	[0.58; 3.13]	*0.396*	1.17	[0.63; 2.06]	*0.601*

^a^For patients with primary lung cancer diagnosed 1 and 3 months ago (multivariate logistic regression model; reduced model)

CI: confidence interval; N: number of subjects; OR: odds ratio

Note: Significant *p*-value are in bold

### Characteristics of the first therapeutic strategy according to patient’s vital status and in dead patients at 1 and 3 months

Table 4 presents the main characteristics of the first therapeutic strategy according to patients’ vital status at 1 and 3 months after diagnosis.

**Table 4:** First therapeutic strategy according to patients’ vital status^a^ (*N* = 6,981)

	Vital status at					
	1 month			3 months		
	Alive	Dead		Alive	Dead	
	*N* = 6,303	*N* = 678	*p-value*	*N* = 5,360	*N* = 1,621	*p-value*
At least one therapy	***n*** **= 6,303**	***n*** **= 678**		***n*** **= 5,360**	***n*** **= 1,621**	
Yes, n (%)	6,177 (98.0)	559 (82.4)		5,273 (98.4)	1,463 (90.3)	
			***<0.001***			***<0.001***
No, n (%)	126 (2.0)	119 (17.6)		87 (1.6)	158 (19.7)	
At least one cancer therapy	***n*** **= 6,303**	***n*** **= 678**		***n*** **= 5,360**	***n*** **= 1,621**	
Yes, n (%)	5,789 (91.8)	241 (35.5)		5,102 (95.2)	928 (57.2)	
			***<0.001***			***<0.001***
No, n (%)	514 (8.2)	437 (64.5)		258 (4.8)	693 (42.8)	
At least one cancer therapy, exclusively	***n*** **= 6,303**	***n*** **= 678**		***n*** **= 5,360**	***n*** **= 1,621**	
Yes, n (%)	5,740 (91.1)	225 (33.2)		5,084 (94.9)	881 (54.3)	
			***<0.001***			***<0.001***
No, n (%)	563 (8.9)	453 (66.8)		276 (5.1)	740 (45.7)	
Included in a therapeutic trial	***n*** **= 6,303**	***n*** **= 678**		***n*** **= 5,360**	***n*** **= 1,621**	
Yes, n (%)	221 (3.5)	2 (0.3)		209 (3.9)	14 (0.9)	
			***<0.001***			***<0.001***
No, n (%)	6,082 (96.5)	676 (99.7)		5,151 (96.1)	1,607 (99.1)	
Multidisciplinary meeting	***n*** **= 6,296**	***n*** **= 671**		***n*** **= 5,355**	***n*** **= 1,612**	
Yes, n (%)	6,011 (95.5)	488 (72.7)		5,139 (96.0)	1,360 (84.4)	
			***<0.001***			***<0.001***
No, n (%)	285 (4.5)	183 (27.3)		216 (4.0)	252 (15.6)	

^a^1 and 3 months after the diagnosis of primary lung cancer (date of diagnosis = date of histological or cytological sampling)

N or n: number of subjects

Compared with the other patients, patients who early died less frequently received at least 1 cancer therapy (curative surgery incl.) (*p* < 0.001). At least 1 cancer treatment was prescribed in 35.5 % of patients who died within 1 month and 57.2 % of patients who died within 3 months. Overall 14 patients who early (0.9 %) died were included in a clinical trial. The first therapeutic strategy was less frequently discussed during a multidisciplinary meeting in patients who early died than in the other patients (*p* < 0.001).

In patients who early died, radiofrequency was exceptional (≤0.2 %) and curative surgery rare (≤3 %). Radiotherapy was infrequent (9.7 % and 18.5 % of patients who died within 1 and 3 months, respectively) and quasi-exclusively palliative (e.g., treatment of cerebral metastasis). Patients with radiotherapy who died within 1 or 3 months had virtually no curative radiotherapy (0.0 and 0.6 %). Respectively, 27.6 % and 44.5 % of patients who died within 1 and 3 months received chemotherapy. Chemotherapy was palliative in almost all patients (97.3 % and 97.9 %, respectively), and only 71 patients received a targeted therapy. Respectively, 49.3 % and 35.9 % of patients who died within 1 and 3 months received supportive care.

### Characteristics of patients without cancer therapy

**Fig. 1:**
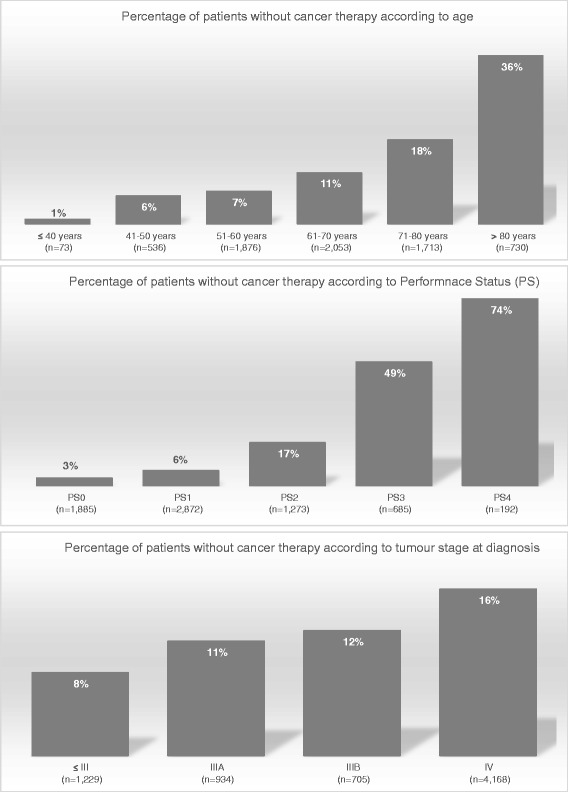
Percentage of patients without cancer therapy according to age, performance status, and tumour stage

Of the 951 patients without cancer therapy, respectively, 437 (46.0 %) and 693 (72.9 %) were dead 1 and 3 months after the diagnosis. The percentage of patients who received no cancer therapy increased with increasing age, PS, and tumour stage (Fig. 1).

The main characteristics of the 951 patients without cancer treatment are presented in Table 5 according to vital status at 1 and 3 months.

**Table 5:** Characteristics of patients without cancer therapy according to their vital status^a^ (*N* = 951)

		Vital status at					
		1 month			3 months		
		Alive	Dead		Alive	Dead	
	N	*N* = 514	*N* = 437	*p-value*	*N* = 258	*N* = 693	*p-value*
Sex		***n*** **= 514**	***n*** **= 437**	***0.071***	***n*** **= 258**	***n*** **= 693**	*0.911*
Men, n (%)	733	384 (74.7)	349 (79.9)		200 (77.5)	533 (76.9)	
Women, n (%)	218	130 (25.3)	88 (20.1)		58 (22.5)	160 (23.1)	
Age		***n*** **= 514**	***n*** **= 437**	***<0.001*** ^c^	***n*** **= 258**	***n*** **= 693**	***<0.001***
≤40 years, n (%)	1	0 (0)	1 (0.2)		0 (0)	1 (0.1)	
41-50 years, n (%)	31	14 (2.7)	17 (3.9)		4 (1.6)	27 (3.9)	
51-60 years, n (%)	135	54 (10.5)	81 (18.5)		24 (9.3)	111 (16.0)	
61-70 years, n (%)	216	101 (19.6)	115 (26.3)		46 (17.8)	170 (24.5)	
71-80 years, n (%)	302	179 (34.8)	123 (28.1)		101 (39.1)	201 (29.0)	
>80 years, n (%)	266	166 (32.3)	100 (22.9)		83 (32.2)	183 (26.4)	
Age		***n*** **= 514**	***n*** **= 437**	***<0.001***	***n*** **= 258**	***n*** **= 693**	***<0.001***
Mean (SD)		74.2 (10.9)	70.1 (11.4)		75 (9.9)	71.3 (11.7)	
Median (Q1-Q3)		76.5 (67-82)	71 (61-80)		77 (69-82)	72 (62-81)	
Body mass index (BMI; kg/m^2^)		***n*** **= 414**	***n*** **= 337**	***0.863***	***n*** **= 221**	***n*** **= 530**	***0.015***
Mean (SD)		23.6 (5.1)	23.5 (4.7)		24.4 (5.4)	23.2 (4.6)	
Median (Q1-Q3)		23.1 (20.1-26.8)	23.4 (20.2-26.4)		24 (20.6-27.4)	23 (19.9-26.2)	
Weight loss within the last 3 months		***n*** **= 490**	***n*** **= 394**	***<0.001***	***n*** **= 249**	***n*** **= 635**	***<0.001***
No, n (%)	279	183 (37.3)	96 (24.4)		115 (46.2)	164 (25.8)	
Yes, n (%)	605	307 (62.7)	298 (75.6)		134 (53.8)	471 (74.2)	
If yes,		***n = 298***	***n = 282***	***0.224***	***n = 130***	***n = 450***	***0.010***
<5 kg, n (%)	193	108 (36.2)	85 (30.1)		56 (43.1)	137 (30.4)	
5-10 kg, n (%)	233	118 (39.6)	115 (40.8)		56 (43.1)	177 (39.3)	
≥10 kg, n (%)	154	72 (24.2)	82 (29.1)		18 (13.8)	136 (30.2)	
Performance status at diagnosis		***n*** **= 511**	***n*** **= 433**	***<0.001***	***n*** **= 255**	***n*** **= 689**	***<0.001***
0- Fully active, n (%)	65	54 (10.6)	11 (2.5)		42 (16.5)	23 (3.3)	
1- Restricted in heavy physical work, n (%)	179	120 (23.5)	59 (13.6)		78 (30.6)	101 (14.7)	
2- Up and about more than half the day, n (%)	221	127 (24.8)	94 (21.7)		59 (23.1)	162 (23.5)	
3- In bed or sitting in a chair more than half the day, n (%)	336	174 (34.1)	162 (37.4)		63 (24.7)	273 (39.6)	
4- In bed or in a chair all the time, n (%)	143	36 (7.0)	107 (24.7)		13 (5.1)	130 (18.9)	
Smoking status		***n*** **= 505**	***n*** **= 427**	**0.389**	***n*** **= 252**	***n*** **= 680**	*0.174*
Never-smoker, n (%)	121	69 (13.7)	52 (12.2)		37 (14.7)	84 (12.4)	
Former-smoker, n (%)	399	223 (44.2)	176 (41.2)		116 (46.0)	283 (41.6)	
Active smoker, n (%)	412	213 (42.2)	199 (46.6)		99 (39.3)	313 (46.0)	
Tobacco consumption (pack-years) ^b^		***n*** **= 394**	***n*** **= 331**	**0.822**	***n*** **= 198**	***n*** **= 527**	***0.025***
Mean (SD)		47.2 (26.3)	46.1 (22.4)		50 (27.3)	45.5 (23.4)	
Median (Q1-Q3)		45 (30-60)	42 (30-59)		50 (30-60)	42 (30-55)	
Tobacco duration (years) ^b^		***n*** **= 340**	***n*** **= 280**	***0.016***	***n*** **= 172**	***n*** **= 448**	***0.012***
Mean (SD)		41.9 (13.5)	39.7 (12)		42.9 (14.1)	40.1 (12.3)	
Median (Q1-Q3)		40 (31.8-50)	40 (30-50)		43 (35-50)	40 (30-50)	
Histology		***n*** **= 514**	***n*** **= 437**		***n*** **= 258**	***n*** **= 693**	
Small-cell carcinoma, n (%)	101	26 (5.1)	75 (17.2)	***<0.001***	5 (1.94)	96 (13.9)	***<0.001***
Adenocarcinoma, n (%)	394	215 (41.8)	179 (41.0)	*0.838*	96 (37.2)	298 (43.0)	*0.124*
Squamous-cell carcinoma, n (%)	297	201 (39.1)	96 (22.0)	***<0.001***	117 (45.4)	180 (26.0)	***<0.001***
Large-cell carcinoma, n (%)	155	70 (13.6)	85 (19.5)	***0.019***	38 (14.7)	117 (16.9)	*0.483*
Adenocarcinoma *in situ*, n (%)	5	2 (0.4)	3 (0.7)	*0.855*	2 (0.8)	3 (0.4)	*0.885* ^c^
Carcinoid tumour, n (%)	3	2 (0.4)	1 (0.2)	*1.000* ^c^	1 (0.4)	2 (0.3)	*1.000* ^c^
Other, n (%)	11	7 (1.4)	4 (0.9)	*0.736*	3 (1.2)	8 (1.2)	*1.000* ^c^
Genomic mutation		***n*** **= 508**	***n*** **= 430**		***n*** **= 254**	***n*** **= 684**	
Explored, n (%)	195	110 (21.7)	85 (19.8)	*0.530*	55 (21.7)	140 (20.5)	*0759*
If explored,		***n*** **= 102**	***n*** **= 76**		***n*** **= 50**	***n*** **= 128**	
EGFR mutated, n (%)	12	8 (7.84)	4 (5.26)	*0.706*	4 (8)	8 (6.3)	*0.932*
Stage (7 edition)		***n*** **= 502**	***n*** **= 432**	***<0.001***	***n*** **= 252**	***n*** **= 682**	***<0.001***
Stage ≤ IIB, n (%)	94	79 (15.7)	15 (3.5)		65 (25.8)	29 (4.3)	
Stage IIIA, n (%)	99	75 (15.0)	24 (5.6)		50 (19.8)	49 (7.2)	
Stage IIIB, n (%)	88	51 (10.2)	37 (8.6)		33 (13.1)	55 (8.1)	
Stage IV, n (%)	653	297 (59.2)	356 (82.4)		104 (41.3)	549 (80.5)	

^a^1 and 3 months after the diagnosis of primary lung cancer (date of diagnosis = date of histological or cytological sampling);

^b^smokers (former or active) only;

^c^Fisher test as Chi^2^-test conditions were not respected

N or n: number of subjects; Q1-Q3: First and third quartiles; SD: standard deviation

Note: Significant *p*-value are in bold

Compared with the other patients with no cancer therapy, patients who early died were significantly younger (*p* < 0.001), they more frequently reported recent weight loss (*p* < 0.001), and more frequently reported a PS of 3 or 4 (*p* < 0.001). Respectively, 107 (82.3 %) and 130 (90.9 %) of the 143 patients with PS4 died within 1 and 3 months. Patients who early died more frequently had a small-cell carcinoma and less frequently presented with squamous-cell carcinoma (*p* < 0.001) than the other patients with no cancer therapy. They also more frequently had a stage IV tumour: respectively, 318 (54.5 %) and 549 (84.0 %) of the 653 patients with stage IV tumour died within 1 and 3 months. The percentage of patients who early died rose with PS and tumour stage increase but not with age increase.

## Discussion

The results of this study showed that in 2010, in France, about 1 to 10 patients managed for primary lung cancer in the respiratory department of a general hospital died within 1 month and about 1 to 4 died within 3 months after lung cancer diagnosis. They also showed that patients who died within 1 or 3 months following lung cancer diagnosis were older and frailer (based on PS and weight loss before diagnosis) than the other patients and more frequently had a lung cancer at advanced stage. Finally, they pointed out that most of these patients had no cancer therapy and most of patients without cancer therapy early died.

### Quantification of early death

To the best of our knowledge, this study is one of the first aimed to evaluate the percentage of patients with lung cancer who early died in clinical practice regardless of their characteristics (e.g., age, PS) and tumour stage. It shows that a significant proportion of patients died within 1 (9.7 %) or 3 months (23.2 %) following diagnosis. In the comparable KBP-2000-CPHG study, performed 10 years ago in French general hospitals, the 3-month mortality was very close (22.1 %) indicating the lack of improvement in 10 years. However, 3-month mortality rate in our study was lower than that recently reported by O’Dowd et al. in UK [3], 30 %. The 3-month mortality among patients with inoperable non-small cell lung cancer who developed respiratory failure was also reported in a retrospective study and was very high in this population (94.4 %) [5].

### Main characteristics of patients who early died

Univariate analyses showed that patients who early died had greater PS, lower BMI, and greater recent weight loss than the other patients. Patients also more frequently reported stage III or IV cancer. Age, sex, PS, histology type, and cancer stage were included in the 4-year mortality score developed and validated in 2006 using data from KBP-2000-CPHG study [7].

In the present study, we included recent weight loss. In the literature, weight loss (>10 %) is a well-known bad prognostic factor [8, 9]. Our results tended to indicate that weight loss was a risk factor of early death and also possibly a warning factor.

Early mortality did not seem to depend on sex and was poorly associated with histology type. Small-cell carcinoma was an independent risk-factor of mortality at 1 month but a protective-factor at 3 months. This apparent discrepancy possibly reflects the good initial response to chemotherapy of small-cell lung cancers [10]. All in all, the most important parameter in early mortality is probably the cancer therapy that is actually performed, in particular chemotherapy, and PS at diagnosis which determines treatment [11, 12].

In the recent UK study by O’Dowd et al. [3], high rate of pre-diagnosis consultations, social deprivation and rural residence are associated factors with early death. These indicators were not registered in our study.

### Patients who early died usually did not receive cancer therapy

Most of the patients who early died did not receive any specific cancer therapy, especially the oldest. Patients who early died more frequently had tumour at advanced stage and non-operable status. They get less curative treatment (surgery, radiofrequency, radiotherapy) and more palliative treatment (chemotherapy, radiotherapy). Despite the French National Cancer Institute developed in 2010 a national program for mutation screening in lung cancer giving us result in 7 days (4 to 25 days), this population get less mutation screening and targeted therapy prescribed. Also, clinical trial access was lower. However, these patients benefits from a multidisciplinary meeting discussion as well as the others. The youngest patients get more than the oldest a cancer treatment.

Probably because patients were old and had a higher PS (3 or 4), clinicians are helpless. Treatment options cannot easily be used in such patients in particular when they presented with stage III or IV cancer. The decrease in the percentage of patients with cancer therapy with increasing age, PS, and stage confirms this hypothesis.

### What would be the room for improvement?

Prevention, in particular through smoking cessation campaigns, and lung cancer screening are the keys for improving lung cancer mortality rate. Early diagnosis is the key for reducing time to diagnosis and time to treatment and, then, is for great importance to improve early mortality. It can give the patients a chance to get a systemic treatment before deterioration of the general condition (better PS, early stage tumour, less weight loss). Also, it can give the old and frail patients and their family a chance to organise the future. Therefore, physicians should pay attention to the smokers with increased respiratory symptoms and recent weight loss and, rapidly refer to a thoracic oncologist. Reducing early post-operative mortality is for need depending on the resection type (lobectomy versus pneumonectomy, sleeve lobectomy) and the team experience [13]. There is a need for clinical research or trial on the patients with bad prognostic factor (PS, tumour stage, age) and development on palliative care treatment [14]. Lung cancer mutation screening is for great importance in all stages or PSs to give access to targeted therapy even or especially in the poor prognostic population who pays a heavy price to early mortality.

### Strengths and limitations

This study uses a large dataset (7,051 included patients, about 20 % of all lung cancers diagnosed in France in 2010) and gives a true reflect of the lung cancer early mortality in a real-world in France in 2010. However, this is not a registry. This study gives us a view on the management of lung cancer in the general hospital, only. But general hospitals supports about 40–45 % of lung cancer in France. The data completeness was checked as described before [6].

In this analysis, we did not separate NSCLC and SCLC which influence the mortality because we focused on the timing, not on the histology type. In fact, from the clinician point of view, the main problem is that the patient fulfils poor prognostic factors and that he/she has to improve patient’s quality of life and life expectancy.

Finally, the cause of death was unknown but lung cancer was probably the main cause regarding the short interval after diagnosis, the severity of the disease, and the low influence from smoking status. In this study, surgery is pooled with other treatments. However, curative surgery was rare in patients who early died (≤3 %). In addition, we found (data not shown) that 1- and 3-month mortality rates in operated subjects (1.4 % and 3.7 %) were lower than in non-operated subjects (11.4 % and 27.2 %), indicating that perioperative deaths do not explain early mortality. We did not have information on the metastatic site (brain) which could have an influence on the early mortality as well as the site of the palliative radiotherapy performed [12].

## Conclusions

About 10 % of patients with lung cancer deceased within the month following the diagnosis (date of sampling) and 25 % within 3 months. As compared with the other patients, patients who early died were older and frailer (greater PS and recent weight loss) and more frequently presented with stage IV tumour. Also, they presented more frequently with a large-cell or small-cell carcinoma and were less EGFR mutant. Most of these patients did not receive any cancer therapy, probably because clinicians are helpless. There is a need to improve early diagnosis to give the patients a chance to receive a systemic treatment to reduce early mortality and/or to give time to the patients and his/her family to organise the future. Indeed, there is a paucity of clinical data guiding the management of clinicians due to the underrepresentation in clinical trials of old and frail patients. Specific clinical trials using new drugs (targeted therapies) or new therapeutic strategies in old and/or frail patients must be implemented.
